# Pressure-controlled inverse ratio ventilation as a rescue therapy for severe acute respiratory distress syndrome

**DOI:** 10.1186/s40064-016-2440-x

**Published:** 2016-06-14

**Authors:** Toru Kotani, Shinshu Katayama, Satoshi Fukuda, Yuya Miyazaki, Yoko Sato

**Affiliations:** Department of Anesthesiology, Tokyo Women’s Medical University, 8-1 Kawada-cho, Shinjuku-ku, Tokyo, 162-8666 Japan

**Keywords:** Acute respiratory distress syndrome, Inverse ratio ventilation, Pressure controlled ventilation, Ventilator-associated lung injury, Positive end-expiratory pressure

## Abstract

**Purpose:**

Low tidal volume ventilation improves the outcomes of acute respiratory distress syndrome (ARDS). However, no studies have investigated the use of a rescue therapy involving mechanical ventilation when low tidal volume ventilation cannot maintain homeostasis. Inverse ratio ventilation (IRV) is one candidate for such rescue therapy, but the roles and effects of IRV as a rescue therapy remain unknown.

**Methods:**

We undertook a retrospective review of the medical records of patients with ARDS who received IRV in our hospital from January 2007 to May 2014. Gas exchange, ventilation, and outcome data were collected and analyzed.

**Results:**

Pressure-controlled IRV was used for 13 patients during the study period. Volume-controlled IRV was not used. IRV was initiated on 4.4 ventilation days when gas exchange could not be maintained. IRV significantly improved the PaO_2_/FiO_2_ from 76 ± 27 to 208 ± 91 mmHg without circulatory impairment. The mean duration of IRV was 10.5 days, and all survivors were weaned from mechanical ventilation and discharged. The 90-day mortality rate was 38.5 %. Univariate analysis showed that the duration of IRV was associated with the 90-day mortality rate. No patients were diagnosed with pneumothorax.

**Conclusions:**

Pressure-controlled IRV provided acceptable gas exchange without apparent complications and served as a successful bridge to conventional treatment when used as a rescue therapy for moderate to severe ARDS.

## Background

In patients with acute respiratory distress syndrome (ARDS), mechanical ventilation plays a pivotal role in triggering or exacerbating inflammatory responses in the lungs, spreading localized pulmonary inflammation to the systemic circulation and causing multiple organ dysfunction syndrome (Slutsky and Tremblay 1998). Prevention of ventilator-induced lung injury (VILI) is a mainstay of the treatment of ARDS. Low tidal volume ventilation (LTV), which is characterized by limiting both the tidal volume and plateau pressure (Pplat) in combination with positive end-expiratory pressure (PEEP), has been used to minimize alveolar overstretch and repetitive alveolar collapse and reopening, both of which provoke VILI (The acute respiratory distress syndrome network 2000; Amato et al. 1998). A previous study reported that mortality parallels Pplat (Hager et al. 2005), and the safe Pplat threshold is considered to be 30 cmH_2_O.

In some severe cases of ARDS, however, LTV is unable to provide adequate gas exchange (Slutsky and Ranieri 2013). This is partly because limiting the Pplat restricts the amount of PEEP that can be used; additionally, an adequate tidal volume cannot be achieved in the presence of an elevated pleural pressure. Because the mean airway pressure is closely related to oxygenation, a ventilation mode that can raise the mean airway pressure without increasing the Pplat could be a useful means of maintaining oxygenation (Yanos et al. 1998). Airway pressure release ventilation (APRV) (Modrykamien et al. 2011) and high-frequency oscillatory ventilation both aim to achieve these goals and have been recommended for severe cases of ARDS caused by influenza A-H1N1 pneumonia. Although extracorporeal membrane oxygenation (ECMO) is a suggested substitute to avoid VILI and can benefit patients with severe ARDS (Webb et al. 2009; Peek et al. 2009), its clinical application is limited because the technique is very resource-intensive (Peek et al. 2009). Therefore, a ventilation modality that can be implemented for severe ARDS in the clinical setting of any facility is warranted.

Inverse ratio ventilation (IRV) is another technique that uses the same principles to improve oxygenation (Cole et al. 1984; Lain et al. 1989; Gurevitch et al. 1986; Tharratt et al. 1988; Abraham and Yoshihara 1989) as other rescue therapies and can be undertaken with the majority of intensive care unit (ICU) ventilators at no additional cost. Despite some promise, previous studies have found IRV to have little or no benefit in patients with severe ARDS (Mercat et al. 1993; Lessard et al. 1994; Mancebo et al. 1994; Mercat et al. 1997; Zavala et al. 1998). However, these studies were conducted more than 20 years ago without the concept of lung protection and designed to compare the short-term effects on gas exchange, hemodynamic parameters, and static compliance. The aim of this study is to assess whether IRV is feasible as a rescue therapy for life-threatening gas exchange failure when conventional ventilation modes could not maintain. We carried out a retrospective review of medical records and investigated the physiological data, the outcomes, and complications associated with IRV.

## Methods

### Ethics statement

The study protocol was approved by our institutional ethics committee (reference number 2721).

### Study design and patient cohort

This was a single-center, retrospective, observational study. A retrospective review of the clinical records of all patients with ARDS treated in the ICU of our institution from January 2007 to May 2014 was undertaken. All patients diagnosed with ARDS according to the Berlin definition (Ranieri et al. 2012) and ventilated with IRV were included in the analysis.

### Data

We recorded age, sex, body weight, underlying diseases, presumed cause of ARDS, indications for IRV, acid–base balance, serum lactate concentration, acute physiological and chronic health evaluation (APACHE) II score at ICU admission, mechanical ventilation parameters, and Murray score before switching to IRV. The arterial partial pressure of oxygen to fraction of inspired oxygen (PaO_2_/FiO_2_, P/F) was calculated, and the timing of initiation of IRV, duration of IRV, total duration of ventilation, and 28- and 90-day mortality were also collected. Arterial blood pressure and heart rate before and after the initiation of IRV and the incidence of pneumothorax were collected to assess the adverse effects.

### Mechanical ventilation and sedation strategies

Mechanical ventilation was initiated with pressure-controlled assist-control ventilation (ACV) according to our procedures for acute hypoxemic respiratory failure. Briefly, the expiratory tidal volume and the Pplat were limited <8 ml/kg predicted body weight and 30 cmH_2_O, respectively. When refractory hypoxemia [arterial oxygen saturation measured by pulse oximetry (SpO_2_) of <90 %] persisted even when the Pplat reached 30 cmH_2_O with a fraction of inspired oxygen of >0.6, the ventilator mode was switched to APRV or IRV with the aim of achieving an adequate mean airway pressure and thus maintaining acceptable and stable oxygenation. The APRV parameters were set according to a previous review (Habashi 2005). When APRV fails to maintain gas exchange, it was converted to IRV. When commencing IRV, the inspiratory to expiratory time ratio was fixed at 2:1, and the tidal volume was initially set at 4–7 ml/kg predicted body weight. Additionally, PEEP was applied to maintain a mean airway pressure of 2–4 cmH_2_O higher than that of the previous ventilation mode and was increased incrementally until acceptable gas exchange was achieved. The respiratory frequency, peak inspiratory pressure (PIP), and PEEP were adjusted to avoid respiratory acidosis and maintain a pH of >7.25. Cardiac ultrasonography was performed daily to evaluate the right ventricular function. The Richmond agitation-sedation scale score (Sessler et al. 2002) was maintained at −3 to −4 during IRV. Sedation had not been interrupted until IRV was terminated.

### Statistical analysis

Statistical analyses were performed using JMP 9 (SAS Institute Inc., Cary, NC, USA). The significance of the categorical variables was calculated using Fisher’s exact test. A t test was used to compare quantitative variables, which were presented as either mean ± standard deviation or proportion (%). Univariate analysis was performed to screen variables associated with the 90-day mortality rate. A p value of <0.05 was considered statistically significant.

## Results

Of 116 patients with ARDS screened during the study period, 13 patients had received IRV and were included in the analysis; their characteristics are summarized in Table 1. The APACHE II score at ICU admission was 29.4 ± 8.7. Two patients had been diagnosed with chronic obstructive pulmonary disease and were being treated. The indication for IRV was refractory hypoxemia in all patients. The worst P/F before the initiation of IRV was 76 ± 27 mmHg. After the initiation of IRV, patient 1 underwent decompression laparotomy to resolve grade IV abdominal compartment syndrome due to large bowel obstruction, and patients 10 and 12 received prone positioning. The ventilation modes prior to IRV were ACV (n = 8) or APRV after failure of ACV (n = 5). The average Murray score calculated in eight patients ventilated with ACV was 3.2 ± 0.5. The Murray score was not calculated in the patients ventilated with APRV because only release volume (not tidal volume) was measured with this modality. IRV was started within 72 h after the start of mechanical ventilation. Patients 3 and 13 had late-onset ARDS, and IRV was started on ventilator day 14 and 21, respectively. Patient 12 was evaluated on ventilator day 4, and IRV was started on that day. Volume-controlled IRV was not used in the study.

**Table 1:** Patient characteristics

Patient	Age	Sex	Body weight (kg)	APACHE II at ICU admission	Cause of ARDS	COPD	Reason of IRV	Ventilator mode before IRV	Murray score before IRV	Additional pulmonary protection
1	79	F	49	36	Septic shock/ACS	−	Hypoxemia/acidemia	APRV	NA	Decompression laparotomy
2	69	M	38	26	Sepsis	+	Hypoxemia	ACV	3.25	
3	62	M	65	23	Pneumonia/sepsis	−	hypoxemia	ACV	3.5	
4	81	M	60	28	Pneumonia	−	Hypoxemia	APRV	NA	
5	77	M	58	46	Pneumonia	−	Hypoxemia/acidemia	ACV	3.5	
6	69	M	43	39	Septic shock/liver abscess	−	Hypoxemia	ACV	3.0	
7	69	F	47	24	Septic shock	−	Hypoxemia	ACV	4.0	
8	70	M	60	14	Sepsis	+	Hypoxemia/acidemia	ACV	3.0	
9	62	M	60	40	Septic shock	−	Hypoxemia/acidemia	ACV	2.5	
10	77	F	51	31	Septic shock	−	Hypoxemia/acidemia	APRV	NA	Prone position
11	68	M	48	26	Sepsis	−	Hypoxemia/acidemia	APRV	NA	
12	57	M	81	26	Pneumonia/septic shock	−	Hypoxemia/acidemia	APRV	NA	Prone position
13	72	M	53	23	Pneumonia/sepsis	−	Hypoxemia/acidemia	ACV	2.75	

*APACHE II* acute physiological and chronic health evaluation II score, *ARDS* acute respiratory distress syndrome, *ICU* intensive care unit, *COPD* chronic obstructive pulmonary disease, *IRV* inverse ratio ventilation, *ACS* abdominal compartment syndrome, *ACV* assist-control ventilation, *APRV* airway pressure release ventilation, *NA* not available

### Ventilator parameters and effects of IRV on gas exchange

The mean Pplat and PEEP in ACV before IRV were 29.4 ± 3.3 and 12.4 ± 3.5 cmH_2_O, respectively. The mean highest PEEP in APRV before IRV was 29.0 ± 4.0 cmH_2_O. The ventilation frequency during IRV was 31–57 breaths/min. The changes in Pplat, PEEP and ventilation frequency over time are shown in Table 2. The Pplat significantly increased after IRV (p = 0.00042).

**Table 2:** Ventilator parameters, hemodynamics, and the dose of noradrenaline before and after IRV

	Pplat^a^ (cmH_2_O)	PEEP (cmH_2_O)	Frequency (breaths/min)	Systolic BP (mmHg)	Mean BP (mmHg)	Noradrenaline (µg/kg/min)
Before IRV	26 ± 6	11 ± 5^b^	17 ± 7	103 ± 21	76 ± 16	0.135 ± 0.216
1 h after IRV	37 ± 6	20 ± 5	40 ± 9	108 ± 13	83 ± 14	0.130 ± 0.150
6 h after IRV	35 ± 4	18 ± 4	39 ± 9	112 ± 20	81 ± 12	0.110 ± 0.142
12 h after IRV	34 ± 3	17 ± 3	38 ± 10	119 ± 21	84 ± 15	0.102 ± 0.133
24 h after IRV	34 ± 4	17 ± 3	41 ± 13	120 ± 14	85 ± 10	0.076 ± 0.100

Data are presented mean ± SD

*Pplat* plateau pressure, *PEEP* positive end-expiratory pressure, *BP* blood pressure, *IRV* inverse ratio ventilation

^a^Pplat is calculated regardless of the modality

^b^Averaged PEEP is calculated in the patients ventilated with ACV

**Fig. 1:**
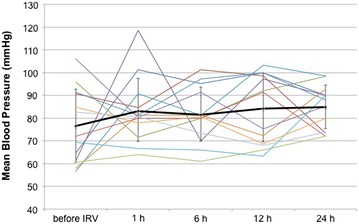
Changes in mean blood pressure in the first 24 h. The *bold line* and *error bar* express average and standard deviation, respectively

Hypoxemia and acidosis was improved over time after IRV (Table 3). The time courses of P/F in all cases were shown in Fig. 1. The P/F significantly increased to 208 ± 91 mmHg within the first 24 h after IRV compared with before IRV (p = 0.0000025).

**Table 3:** Arterial blood gas analysis before and after IRV

	pH	PaO_2_/FiO_2_ (mmHg)	PaCO_2_ (mmHg)
Before IRV	7.29 ± 0.12	100 ± 62	47 ± 13
1 h after IRV	7.31 ± 0.09	144 ± 112	47 ± 16
6 h after IRV	7.29 ± 0.09	157 ± 92	46 ± 14
12 h after IRV	7.35 ± 0.08	192 ± 94	41 ± 13
24 h after IRV	7.36 ± 0.06	196 ± 90	41 ± 9

### Adverse events associated with IRV and the mortality rate

**Fig. 2:**
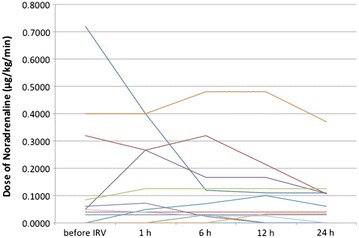
The doses of noradrenaline in the first 24 h. Twelve of thirteen patients received noradrenaline infusion

Five patients were in shock status when IRV was introduced. Patient 1 became hemodynamically unstable on initiation of IRV and was successfully treated with volume resuscitation and administration of vasopressors. The remaining four patients did not require any additional resuscitation. Mean blood pressure was stable in the first 24 h after the initiation of IRV (Fig. 1). Noradrenaline was used in 12 of 13 patients. The doses of noradrenaline were decreased or unchanged in the first 24 h after the initiation of IRV (Fig. 2) and no further circulatory events were observed. All patients were sedated with a combination of fentanyl, dexmedetomidine, and propofol to facilitate mechanical ventilation. Pneumothorax was not observed, although no patients were paralyzed.

The 28- and 90-day mortality rates were 30.8 and 38.5 %, respectively. All survivors were switched to APRV to maintain their mean airway pressure and weaned from mechanical ventilation, whereas all nonsurvivors were ventilated with IRV to the end. Age, the APACHE II score, the ventilation days before IRV, and the duration of IRV in survivors and non-survivors were shown in Table 4. Univariate analysis showed that the duration of IRV was associated with the 90-day mortality rate (p = 0.009).

**Table 4:** Risk factors for 90-day mortality rate

	Total (n = 13)	Survivors (n = 8)	Nonsurvivors (n = 5)	p value
Age (years)	70 ± 7	72 ± 8	67 ± 6	0.221
Male no. (%)	10 (77 %)	5 (63 %)	5 (100 %)	0.118
APACHE II at ICU admission	29.4 ± 8.7	30.3 ± 10.3	28.0 ± 6.3	0.670
MV before IRV (days)	4.4 ± 6.1	3.5 ± 4.4	5.8 ± 8.5	0.529
IRV duration (days)	10.5 ± 9.6	5.5 ± 4.2	18.6 ± 10.6	0.009

*APACHE* acute physiological and chronic health evaluation, *ICU* intensive care unit, *MV* mechanical ventilation, *IRV* inverse ratio ventilation

## Discussion

We found that IRV was started immediately after the failure of LTV or APRV, and improved oxygenation without major complications such as cardiovascular deterioration and pneumothorax. The Murray scores of patients in our cohort, who fulfilled the criteria for ECMO, reflect the severity of ARDS. There was no difficulty weaning back to a more conventional mode of ventilation in survivors. Mortality was acceptable compared with a recent report (Ranieri et al. 2012). These findings suggest that IRV has the potential to be an effective and safe option for temporarily maintaining gas exchange for refractory hypoxemia that has proved difficult to manage using conventional ventilation modes. IRV is feasible as a rescue therapy when ECMO is not available.

In the current study, mechanical ventilation before IRV was performed according to the concept of lung protection, limiting the tidal volume and Pplat. Although the PEEP before IRV was lower than that recommended in a previous randomized controlled trial (The acute respiratory distress syndrome network 2000), the PEEP is often set lower to prevent the Pplat from exceeding the safety threshold (Checkley et al. 2008). This is a clinical limitation of conventional ventilation and confirmed our concern that another modality is required in particular situations.

It is suggested that our stepwise protocol for the application of IRV contributed to prevent adverse events. IRV was introduced when the patients were diagnosed as ARDS in the previous studies (Mercat et al. 1993, 1997; Lessard et al. 1994; Mancebo et al. 1994; Zavala et al. 1998). In our study IRV was used as the final option for elevating the mean airway pressure with a smaller increase in the PIP or Pplat because the Pplat had already reached the safety threshold. To facilitate IRV, we chose ventilation parameters that minimized the disadvantages rather than those that provided full therapeutic benefits. When the Pplat and PEEP increased during IRV, stepwise titration was performed to obtain the lowest appropriate pressure. Although the Pplat exceeded the safety threshold of the lung protective strategy in some cases, the stepwise titration procedure maintained a safe minimum pressure, preventing barotrauma. IRV provided acceptable gas exchange to continue the treatment for patients at life-threatening risk, and this was the aim of IRV. We tried to minimize the duration of IRV, and as soon as it was feasible the mode was changed to APRV, which reliably maintained a mean airway pressure during IRV and was easier to synchronize with the patient’s own respiratory pattern. These factors may account for the low incidence of adverse events and relatively low mortality rate.

One of the mechanisms underpinning the improvement in oxygenation is thought to be promotion of alveolar recruitment (Marini and Ravenscraft 1992) due to the longer inspiratory time. Another mechanism is the prevention of derecruitment due to the shorter expiratory time. Insufficient alveolar pressure may cause tidal recruitment/derecruitment of the alveoli, exposing the regions to shear stress (Ochiai 2015). A recent animal study demonstrated that IRV minimized cyclic recruitment and derecruitment of atelectasis and improved oxygenation compared with the conventional mode (Boehme et al. 2015). This was accompanied by redistribution of ventilation from the nondependent to dependent lung regions (Kotani et al. 2016). Because the ventilatory frequency during IRV was 31–57 breaths/min in our cohort, the very short expiratory time contributed to stabilization of the alveoli in accordance with the longer inspiratory time.

IRV is associated with a risk of developing VILI because of the higher pressure required to commence IRV. It is well recognized that IRV can cause hemodynamic instability as a consequence of an increased intrinsic PEEP and mean airway pressure (Cole et al. 1984). However, we did not observe these adverse events in the current study. Hemodynamic compromise can be prevented or ameliorated by the incremental introduction of higher ventilation pressures, volume resuscitation, or vasopressor therapy; this is the approach we adopted in one of the patient who experienced hemodynamic instability in our study. We found that a higher PEEP caused hypercapnia, but this was ameliorated by increasing the ventilator frequency. Therefore, the overall benefits of IRV appear to outweigh its disadvantages.

Our study has several limitations. It was a single-center, retrospective, case-series study. Additionally, the patients’ clinical condition is not common, and the sample size was therefore small. Whether severe complications develop during IRV depends in major part on the clinical experience of the staff. There were several uncontrolled factors having an impact on the outcomes, such as patient background, fluid balance, and the timing of IRV introduction and termination. Switching to IRV would likely have occurred earlier in the patients’ clinical course than in previous studies. We did not measure or record lung parameters (e.g., mean airway pressure and intrinsic PEEP) or esophageal pressure. An inspiratory to expiratory ratio of 2:1 was used in the study, but the effect of this ratio on the outcomes is unclear. We were unable to establish whether IRV reduced lung injury because we did not measure the biomarkers associated with VILI and did not routinely perform lung biopsy. Finally, the feasibility of volume-controlled IRV is unknown because we did not use it.

## Conclusions

Pressure-controlled IRV provided acceptable oxygenation without major complications, suggesting that pressure-controlled IRV is feasible as a rescue therapy in patients with ARDS that do not respond to conventional treatment. Further studies are warranted to confirm the efficacy and safety of pressure-controlled IRV as a rescue therapy for moderate to severe ARDS.

## Abbreviations

LTV: low tidal volume ventilation
ARDS: acute respiratory distress syndrome
IRV: inverse ratio ventilation
VILI: ventilator-induced lung injury
Pplat: plateau pressure
PEEP: positive end-expiratory pressure
APRV: airway pressure release ventilation
ECMO: extracorporeal membrane oxygenation
ICU: intensive care unit
APACHE: acute physiological and chronic health evaluation
P/F: arterial partial pressure of oxygen–fraction of inspired oxygen
ACV: assist-control ventilation
SpO_2_: arterial oxygen saturation measured by pulse oximetry
PIP: peak inspiratory pressure
